# Supplementary material for: A Likelihood Approach for Real-Time Calibration of Stochastic Compartmental Epidemic Models
Source: PLoS Comput Biol. 2017 Jan 17;13(1):e1005257. doi: 10.1371/journal.pcbi.1005257 (PMC5240920; doi:10.1371/journal.pcbi.1005257)
Supplement: S1 File — (TAR.GZ) [file pcbi.1005257.s014.tar.gz › HSPH_Online-SI-Revision/PF08/n100-mild/n100-mild_PF_table8.pdf]

1	R0	{1.24125, 3.38982}	{1.92232, 18.5076}	{{1.92212, 18.4814}, {1.92398, 18.5249}}	{54.9422, 445.977}	inf	161.	11534.7	{11511.8, 11548.6}	7064.41	1.85518	newly	287.	4046.72	{3969., 4113.}	1310.01	1.14918	Reff	0.815836	0.615174	{0.614822, 0.615522}	24.5959	ar	34926.	93903.3	{93685., 94093.}	168.864
2	R0	{1.29554, 4.51878}	{1.70922, 14.074}	{{1.70074, 14.0131}, {1.71654, 14.1108}}	{31.9313, 211.456}	inf	262.	6585.6	{6555.4, 6629.1}	2413.59	1.40029	newly	367.	3006.33	{2945., 3063.}	719.163	0.91332	Reff	0.75267	0.664657	{0.661472, 0.667925}	11.6934	ar	42828.	88418.9	{88065., 88743.}	106.451
3	R0	{1.2443, 3.64292}	{1.87447, 17.0942}	{{1.869, 17.0232}, {1.8818, 17.153}}	{50.6449, 369.244}	inf	255.	10251.3	{10188.2, 10297.5}	3920.13	1.60423	newly	387.	3901.99	{3832., 3966.}	908.266	1.00354	Reff	0.790168	0.658146	{0.65677, 0.659211}	16.708	ar	37087.	95606.7	{95321., 95880.}	157.79
4	R0	{1.38465, 3.43331}	{3., 20.}	{{3., 20.}, {3., 20.}}	{116.661, 482.528}	inf	64.	6657.55	{6625.18, 6690.54}	10302.4	2.01713	newly	86.	2046.96	{2002., 2090.}	2280.19	1.37655	Reff	0.700356	0.256784	{0.247057, 0.260455}	63.3352	ar	49511.	109043.	{107898., 109990.}	120.24
5	R0	{1.25409, 4.1068}	{1.81586, 15.303}	{{1.81497, 15.2706}, {1.81734, 15.3199}}	{44.7947, 272.627}	inf	297.	7355.14	{7344.37, 7371.78}	2376.48	1.39383	newly	386.	3074.92	{3015., 3128.}	696.611	0.901202	Reff	0.780997	0.597853	{0.597136, 0.598251}	23.4501	ar	38571.	86316.1	{86061., 86536.}	123.785
6	R0	{1.28821, 4.2016}	{1.96251, 17.5842}	{{1.96039, 17.5399}, {1.96371, 17.6288}}	{52.3439, 318.512}	inf	242.	8166.67	{8136.44, 8204.56}	3274.66	1.52823	newly	315.	2988.44	{2928., 3046.}	848.711	0.977083	Reff	0.761538	0.576334	{0.57488, 0.577547}	24.3197	ar	41468.	93006.8	{92826., 93277.}	124.358
7	R0	{1.24369, 2.56007}	{2.89171, 20.}	{{2.89088, 20.}, {2.89279, 20.}}	{132.511, 681.229}	inf	25.	9794.63	{9789.76, 9801.79}	39078.5	2.59305	newly	53.	3024.16	{2960., 3077.}	5605.96	1.75627	Reff	0.764869	0.280008	{0.279666, 0.280346}	63.3914	ar	38603.	103226.	{103138., 103307.}	167.403
8	R0	{1.232, 3.53942}	{1.88463, 17.7813}	{{1.8787, 17.6616}, {1.88846, 17.9616}}	{52.9731, 402.379}	inf	101.	9416.44	{9336.22, 9525.16}	9223.21	1.96955	newly	163.	3414.83	{3357., 3474.}	1994.99	1.32114	Reff	0.809486	0.579333	{0.578169, 0.58058}	28.432	ar	36875.	94878.4	{94397., 95478.}	174.572
9	R0	{1.36262, 3.85631}	{2.49022, 18.0849}	{{2.48302, 18.0321}, {2.4952, 18.1497}}	{82.7522, 368.968}	inf	139.	7094.69	{7061.93, 7163.75}	5004.09	1.70791	newly	200.	2421.84	{2372., 2475.}	1110.92	1.08305	Reff	0.708181	0.341859	{0.341359, 0.342317}	51.7272	ar	48410.	98054.3	{97856., 98252.}	102.55
10	R0	{1.28702, 4.67968}	{1.8524, 18.314}	{{1.85183, 18.3061}, {1.85323, 18.3243}}	{43.9294, 291.352}	inf	267.	7468.35	{7457.92, 7474.77}	2697.14	1.44671	newly	358.	2666.05	{2606., 2714.}	644.707	0.871926	Reff	0.767643	0.643043	{0.642507, 0.643525}	16.2315	ar	41092.	86708.4	{86437., 86980.}	111.01
11	R0	{1.35752, 3.47129}	{2.08293, 16.8707}	{{2.07926, 16.8449}, {2.08439, 16.8994}}	{53.4366, 386.006}	inf	143.	8590.81	{8557.63, 8631.88}	5907.56	1.7787	newly	225.	3214.32	{3152., 3272.}	1328.59	1.15486	Reff	0.755256	0.502786	{0.501356, 0.504502}	33.4284	ar	44669.	87483.5	{87303., 87660.}	95.8484
12	R0	{1.35929, 5.83647}	{1.85271, 16.5296}	{{1.84905, 16.4725}, {1.85664, 16.5846}}	{36.2997, 183.212}	inf	598.	7187.62	{7144.79, 7233.27}	1101.94	1.07988	newly	613.	2795.51	{2743., 2846.}	356.038	0.658953	Reff	0.753917	0.592864	{0.591161, 0.59428}	21.3621	ar	46715.	91464.2	{91046., 91831.}	95.7919
13	R0	{1.28019, 3.29908}	{1.89637, 13.1802}	{{1.89305, 13.0988}, {1.89618, 13.2072}}	{48.1318, 299.511}	inf	135.	5290.4	{5217.12, 5329.94}	3818.81	1.59314	newly	244.	2470.73	{2413., 2524.}	912.594	1.00537	Reff	0.759998	0.512429	{0.507409, 0.516485}	32.5749	ar	40941.	87594.	{87090., 88359.}	113.952
14	R0	{1.36661, 3.48318}	{3., 20.}	{{3., 20.}, {3., 20.}}	{119.521, 474.188}	inf	77.	7389.54	{7374.68, 7402.53}	9496.81	1.98213	newly	115.	2297.04	{2239., 2353.}	1897.43	1.30039	Reff	0.705239	0.310738	{0.308428, 0.312548}	55.9386	ar	48672.	115162.	{115084., 115243.}	136.609
15	R0	{1.35865, 3.84123}	{2.21454, 17.1842}	{{2.21329, 17.1651}, {2.21551, 17.2092}}	{62.9954, 347.362}	inf	163.	8010.72	{7983.04, 8051.94}	4814.55	1.69148	newly	228.	2905.8	{2853., 2953.}	1174.47	1.10528	Reff	0.704243	0.438673	{0.437846, 0.439574}	37.7099	ar	48598.	89518.6	{89371., 89670.}	84.2023
16	R0	{1.22879, 3.98329}	{1.80894, 18.0985}	{{1.80374, 18.0654}, {1.81377, 18.1296}}	{47.213, 354.361}	inf	367.	9589.94	{9552.56, 9632.87}	2513.06	1.41715	newly	573.	3525.65	{3459., 3596.}	515.297	0.789034	Reff	0.822699	0.752737	{0.749461, 0.755662}	8.50407	ar	34405.	89015.	{88570., 89422.}	158.727
17	R0	{1.34925, 3.64524}	{2.07614, 13.9825}	{{2.07359, 13.8309}, {2.08076, 14.1112}}	{53.8739, 283.581}	inf	162.	6191.26	{6053.63, 6306.36}	3721.76	1.58218	newly	237.	2711.41	{2653., 2769.}	1044.05	1.05838	Reff	0.721606	0.464422	{0.459511, 0.468875}	35.6125	ar	46871.	94825.3	{94370., 95310.}	102.311
18	R0	{1.33054, 2.25275}	{3., 20.}	{{3., 20.}, {3., 20.}}	{125.472, 787.804}	inf	9.	9449.89	{9440.01, 9458.36}	104899.	3.02118	newly	21.	2915.86	{2867., 2965.}	13785.	2.14251	Reff	0.740885	0.259217	{0.258907, 0.259557}	65.0125	ar	44336.	103069.	{102964., 103147.}	132.472
19	R0	{1.28394, 3.69859}	{1.90501, 18.1487}	{{1.89636, 18.087}, {1.90979, 18.2733}}	{48.3718, 390.694}	inf	204.	10235.3	{10188.4, 10327.5}	4917.28	1.70046	newly	360.	3675.91	{3616., 3735.}	921.086	1.00903	Reff	0.786067	0.639061	{0.63839, 0.63979}	18.7014	ar	39558.	89918.7	{89638., 90163.}	127.309
20	R0	{1.2883, 2.26493}	{3., 20.}	{{3., 20.}, {3., 20.}}	{132.865, 783.029}	inf	22.	10357.1	{10344.1, 10373.2}	46977.8	2.67282	newly	37.	3173.23	{3112., 3236.}	8476.3	1.93325	Reff	0.763434	0.231981	{0.231414, 0.232425}	69.6135	ar	40768.	107842.	{107644., 107958.}	164.525
21	R0	{1.34098, 2.78621}	{2.07339, 12.6903}	{{2.07009, 12.6868}, {2.07663, 12.695}}	{54.6177, 355.469}	inf	39.	5771.19	{5759.6, 5781.66}	14697.9	2.1702	newly	64.	2758.93	{2702., 2810.}	4210.83	1.63451	Reff	0.744378	0.469868	{0.469215, 0.470521}	36.8777	ar	44605.	102375.	{102237., 102497.}	129.515
22	R0	{1.33783, 5.95911}	{1.86652, 17.9083}	{{1.86242, 17.874}, {1.87559, 17.9983}}	{39.5185, 200.52}	inf	686.	7814.69	{7777.36, 7882.42}	1039.17	1.05654	newly	709.	2849.02	{2784., 2896.}	301.836	0.603992	Reff	0.760757	0.650854	{0.646769, 0.653814}	14.4465	ar	45418.	90957.2	{90638., 91477.}	100.267
23	R0	{1.33359, 3.3069}	{2.05483, 15.4413}	{{2.05249, 15.4232}, {2.05694, 15.4607}}	{54.0829, 366.941}	inf	84.	8247.	{8222.96, 8271.28}	9717.86	1.99202	newly	143.	3342.45	{3288., 3393.}	2237.38	1.36869	Reff	0.719592	0.499562	{0.498683, 0.500407}	30.577	ar	46245.	93793.3	{93591., 94004.}	102.818
24	R0	{1.30588, 2.56123}	{1.99564, 20.}	{{1.99461, 20.}, {1.99694, 20.}}	{52.8193, 680.875}	inf	11.	9087.08	{9081.58, 9093.41}	82509.8	2.91703	newly	29.	2928.13	{2868., 2989.}	9997.	2.00413	Reff	0.752997	0.513837	{0.513291, 0.514297}	31.761	ar	42383.	97354.6	{97126., 97589.}	129.702
25	R0	{1.29813, 4.51849}	{1.93826, 20.}	{{1.92973, 20.}, {1.96018, 20.}}	{49.3115, 342.626}	inf	473.	10589.7	{10481.5, 10649.6}	2138.85	1.35002	newly	633.	3481.2	{3405., 3539.}	449.953	0.740281	Reff	0.756044	0.643432	{0.623432, 0.649144}	14.8949	ar	43371.	84989.8	{84611., 85468.}	95.9599
26	R0	{1.33231, 3.99953}	{2.36482, 20.}	{{2.34775, 20.}, {2.37179, 20.}}	{77.498, 400.059}	inf	227.	9555.39	{9525.18, 9614.24}	4109.42	1.62422	newly	332.	3051.41	{2996., 3110.}	819.099	0.963312	Reff	0.747852	0.470295	{0.465737, 0.475443}	37.114	ar	44733.	99601.9	{99127., 99930.}	122.659
27	R0	{1.30555, 4.61445}	{1.94448, 20.}	{{1.93708, 20.}, {1.97668, 20.}}	{48.9397, 333.421}	inf	364.	10620.8	{10511.2, 10657.3}	2817.79	1.46505	newly	467.	3483.36	{3413., 3545.}	645.901	0.872632	Reff	0.764165	0.634184	{0.611068, 0.639254}	17.0095	ar	42624.	85940.3	{85564., 86655.}	101.624
28	R0	{1.26819, 5.34969}	{1.82113, 18.1769}	{{1.81176, 17.92}, {1.83115, 18.5227}}	{43.6009, 239.775}	inf	578.	9288.01	{9129.1, 9488.13}	1506.92	1.20595	newly	683.	3351.43	{3291., 3410.}	390.693	0.690765	Reff	0.806442	0.679879	{0.676485, 0.68321}	15.694	ar	38946.	89948.3	{89557., 90299.}	130.956
29	R0	{1.19925, 3.00394}	{1.91622, 17.4852}	{{1.90567, 17.4258}, {1.92143, 17.5214}}	{59.7846, 482.075}	inf	160.	10476.	{10416.9, 10515.3}	6447.52	1.81607	newly	328.	3913.06	{3849., 3983.}	1093.01	1.07661	Reff	0.835841	0.657671	{0.65527, 0.659257}	21.3163	ar	31727.	92233.3	{91977., 92515.}	190.709
30	R0	{1.35749, 3.75483}	{2.06547, 13.7247}	{{2.06345, 13.7049}, {2.06749, 13.7368}}	{52.1536, 265.52}	inf	77.	4870.22	{4859.05, 4881.48}	6224.96	1.80106	newly	120.	2142.18	{2099., 2188.}	1685.15	1.25162	Reff	0.704592	0.412072	{0.411354, 0.412846}	41.5162	ar	48244.	96819.7	{96690., 96938.}	100.687
31	R0	{1.27131, 4.2161}	{1.79777, 15.8078}	{{1.7958, 15.7937}, {1.7993, 15.8152}}	{41.4104, 274.939}	inf	338.	9316.37	{9297.94, 9322.92}	2656.32	1.44033	newly	482.	3814.03	{3751., 3875.}	691.293	0.898298	Reff	0.797836	0.652473	{0.651956, 0.652645}	18.2196	ar	38299.	90378.9	{90087., 90650.}	135.982
32	R0	{1.31827, 6.18119}	{1.88295, 17.3688}	{{1.84447, 16.8785}, {1.89635, 17.5639}}	{42.8347, 180.994}	inf	827.	7293.02	{7176.87, 7345.97}	781.865	0.945383	newly	844.	2725.46	{2662., 2794.}	222.922	0.509028	Reff	0.757649	0.614279	{0.606203, 0.634398}	18.9231	ar	45262.	90654.4	{89112., 91324.}	100.288
33	R0	{1.20398, 1.35167}	{3., 20.}	{{3., 20.}, {3., 20.}}	{149.174, 1379.65}	inf	3.	11086.1	{11081.7, 11091.5}	369438.	3.56766	newly	6.	3407.87	{3349., 3466.}	56697.8	2.75429	Reff	0.802742	0.246659	{0.246392, 0.246887}	69.2729	ar	33329.	103950.	{103864., 104031.}	211.891
34	R0	{1.32776, 3.146}	{2.89532, 20.}	{{2.89026, 20.}, {2.89937, 20.}}	{118.061, 535.728}	inf	21.	7342.17	{7332.3, 7353.28}	34862.7	2.5436	newly	25.	2265.04	{2205., 2318.}	8960.16	1.95705	Reff	0.733149	0.263338	{0.26211, 0.264309}	64.0813	ar	44872.	99460.7	{99326., 99637.}	121.654
35	R0	{1.279, 1.11336}	{3., 20.}	{{3., 20.}, {3., 20.}}	{134.558, 1696.36}	inf	0.1	12575.5	{12571.1, 12579.8}	1.25754×10^{7}	5.09953	newly	0.1	3880.06	{3818., 3946.}	3.87996×10^{6}	4.5888	Reff	0.754188	0.270497	{0.270278, 0.270722}	64.134	ar	41033.	101971.	{101880., 102064.}	148.511
36	R0	{1.20029, 1.94717}	{2.76423, 20.}	{{2.7633, 20.}, {2.76491, 20.}}	{130.297, 927.132}	inf	45.	12494.	{12487.3, 12500.7}	27664.4	2.44349	newly	117.	3891.1	{3830., 3956.}	3225.73	1.52185	Reff	0.807351	0.328885	{0.328617, 0.329142}	59.2637	ar	33234.	99166.3	{99082., 99268.}	198.388
37	R0	{1.33216, 3.69685}	{2.12607, 17.5394}	{{2.12429, 17.5146}, {2.12855, 17.5926}}	{59.5957, 374.442}	inf	150.	7872.17	{7837.29, 7920.09}	5148.11	1.72	newly	219.	2839.44	{2784., 2901.}	1196.55	1.11273	Reff	0.735299	0.485119	{0.483414, 0.487282}	34.0243	ar	45235.	90765.6	{90558., 90963.}	100.653
38	R0	{1.25434, 2.04314}	{3., 20.}	{{3., 20.}, {3., 20.}}	{139.17, 878.885}	inf	2.	9907.51	{9898.29, 9923.18}	495275.	3.69493	newly	6.	3038.01	{2985., 3088.}	50533.5	2.70439	Reff	0.769437	0.239492	{0.238957, 0.2402}	68.8744	ar	38662.	101917.	{101769., 102042.}	163.611
39	R0	{1.27466, 3.0741}	{2.36889, 16.9269}	{{2.36861, 16.9197}, {2.36974, 16.9359}}	{85.8445, 450.631}	inf	79.	6795.45	{6790.15, 6801.88}	8501.83	1.93459	newly	134.	2471.77	{2413., 2528.}	1744.6	1.26584	Reff	0.765178	0.364044	{0.363743, 0.364235}	52.4236	ar	40181.	103525.	{103426., 103639.}	157.648
40	R0	{1.33045, 2.68154}	{3., 20.}	{{3., 20.}, {3., 20.}}	{125.488, 645.84}	inf	10.	7715.56	{7710.03, 7720.25}	77055.6	2.88737	newly	17.	2359.91	{2302., 2417.}	13781.8	2.14238	Reff	0.719255	0.212909	{0.212615, 0.213259}	70.3986	ar	45951.	105206.	{105045., 105372.}	128.952
41	R0	{1.37003, 2.83984}	{3., 20.}	{{3., 20.}, {3., 20.}}	{118.973, 604.265}	inf	53.	9097.34	{9087.81, 9106.6}	17064.8	2.23464	newly	99.	2803.96	{2752., 2847.}	2732.28	1.45209	Reff	0.701168	0.256668	{0.255743, 0.257443}	63.3942	ar	48926.	101937.	{101769., 102216.}	108.35
42	R0	{1.3003, 2.94885}	{2.64859, 18.1558}	{{2.64724, 18.1431}, {2.64981, 18.1636}}	{103.691, 515.692}	inf	49.	7554.62	{7544.01, 7565.9}	15317.6	2.18802	newly	90.	2542.66	{2498., 2591.}	2725.18	1.451	Reff	0.726881	0.298238	{0.29749, 0.29898}	58.9702	ar	44237.	101098.	{100993., 101206.}	128.537
43	R0	{1.33756, 2.92653}	{3., 20.}	{{3., 20.}, {3., 20.}}	{124.289, 583.403}	inf	64.	9108.41	{9092.96, 9123.89}	14131.9	2.15326	newly	97.	2800.74	{2750., 2858.}	2787.36	1.46046	Reff	0.706272	0.256935	{0.255737, 0.258271}	63.621	ar	47342.	101757.	{101263., 102196.}	114.941
44	R0	{1.35801, 2.92276}	{3., 20.}	{{3., 20.}, {3., 20.}}	{120.911, 584.285}	inf	37.	8429.39	{8421.31, 8437.84}	22682.1	2.35759	newly	67.	2576.68	{2516., 2634.}	3745.79	1.58492	Reff	0.707754	0.216911	{0.215927, 0.217676}	69.3523	ar	47940.	105143.	{104758., 105598.}	119.322
45	R0	{1.31342, 4.02649}	{1.81518, 13.8857}	{{1.81398, 13.8604}, {1.81604, 13.904}}	{38.2029, 244.858}	inf	207.	6208.45	{6185.36, 6228.12}	2899.25	1.47701	newly	308.	2814.03	{2755., 2867.}	813.646	0.960723	Reff	0.755532	0.577024	{0.575982, 0.577903}	23.2628	ar	42995.	86603.8	{86368., 86840.}	101.428
46	R0	{1.37919, 3.75889}	{2.17618, 14.5592}	{{2.17324, 14.5242}, {2.17932, 14.5811}}	{57.7869, 287.328}	inf	174.	6878.92	{6858.01, 6897.71}	3853.4	1.59697	newly	255.	2906.83	{2858., 2955.}	1039.93	1.05684	Reff	0.704463	0.454707	{0.453894, 0.455911}	35.4534	ar	49157.	96410.6	{96269., 96571.}	96.1279
47	R0	{1.37458, 3.56117}	{2.93153, 20.}	{{2.92918, 20.}, {2.9337, 20.}}	{113.267, 461.613}	inf	54.	7841.41	{7828.5, 7852.59}	14421.1	2.162	newly	75.	2412.43	{2364., 2465.}	3116.57	1.50733	Reff	0.710232	0.25711	{0.256115, 0.258029}	63.7992	ar	48478.	101898.	{101782., 102023.}	110.195
48	R0	{1.33393, 3.78296}	{1.95973, 20.}	{{1.95052, 20.}, {1.97472, 20.}}	{46.914, 428.687}	inf	157.	11557.3	{11514.9, 11581.6}	7261.36	1.86696	newly	214.	3806.48	{3725., 3878.}	1678.73	1.25006	Reff	0.740251	0.660608	{0.635392, 0.667668}	10.759	ar	44885.	84942.7	{84471., 85808.}	89.2451
49	R0	{1.24918, 2.39046}	{2.01371, 13.6245}	{{2.0131, 13.6169}, {2.0144, 13.631}}	{61.2027, 469.953}	inf	26.	6048.4	{6042.13, 6055.78}	23163.1	2.36667	newly	69.	2732.8	{2681., 2786.}	3860.58	1.5977	Reff	0.801174	0.498961	{0.498514, 0.499313}	37.7213	ar	35969.	92329.9	{92173., 92511.}	156.693
50	R0	{1.28281, 2.3032}	{3., 20.}	{{3., 20.}, {3., 20.}}	{133.862, 768.357}	inf	27.	7011.07	{6996.35, 7035.24}	25866.9	2.41442	newly	59.	2163.	{2115., 2222.}	3566.1	1.56412	Reff	0.767787	0.272147	{0.270455, 0.273254}	64.5544	ar	40223.	104438.	{103984., 104635.}	159.648
